# ﻿Four new species of Trichomonascaceae (Saccharomycetales, Saccharomycetes) from Central China

**DOI:** 10.3897/mycokeys.90.83829

**Published:** 2022-05-25

**Authors:** Chun-Yue Chai, Wan-Li Gao, Zhen-Li Yan, Feng-Li Hui

**Affiliations:** 1 School of Life Science and Agricultural Engineering, Nanyang Normal University, Nanyang 473061, China Nanyang Normal University Nanyang China; 2 Research Center of Henan Provincial Agricultural Biomass Resource Engineering and Technology, Nanyang 473061, China Research Center of Henan Provincial Agricultural Biomass Resource Engineering and Technology Nanyang China; 3 State Key Laboratory of Motor Vehicle Biofuel Technology, Henan Tianguan Enterprise Group Co., Ltd., Nanyang 473000, China State Key Laboratory of Motor Vehicle Biofuel Technology, Henan Tianguan Enterprise Group Co., Ltd. Nanyang China

**Keywords:** New taxa, phylogenetics, taxonomy, Trichomonascaceae, yeasts

## Abstract

Trichomonascaceae is the largest family of ascomycetous yeast in the order Saccharomycetales. In spite of the extensive body of research on Trichomonascaceae in China, there remain new species to be discovered. Here, we describe four new species isolated from several rotting wood samples from Henan Province, Central China. Phylogenetic analysis of a combined ITS and nrLSU dataset with morphological studies revealed four new species in the Trichomonascaceae: *Diddensiellaluoyangensis*, *Sugiyamaellacylindrica*, *Su.robnettiae*, and *Zygoascusdetingensis*. Clustering in the *Diddensiella* clade, *D.luoyangensis*’ closest neighbour was *D.transvaalensis*. Meanwhile, *Su.cylindrica* clustered in the *Sugiyamaella* clade closest to *Su.marilandica* and *Su.qingdaonensis*. Also clustering in the *Sugiyamaella* clade, *Su.robnettiae* was most closely related to *Su.chuxiongensis*. Finally, *Z.detingensis* occupied a distinct and separated basal branch from the other species of the genus *Zygoascus*. These results indicate a high species diversity of Trichomonascaceae.

## ﻿Introduction

The family of Trichomonascaceae was described by Kurtzman and Robnett (2007) to accommodate the genera *Sugiyamaella* Kurtzman and Robnett, *Trichomonascus* (H.S. Jackson) Kurtzman and Robnett, *Wickerhamiella* van der Walt, *Zygoascus* M.Th. Smith and related anamorphs based on multigene phylogenetic analysis (Kurtzman 2011a). Subsequently, two new genera, *Spencermartinsiella* Péter, Dlauchy, Tornai-Lehoczki, M. Suzuki & Kurtzman and *Diddensiella* Péter, Dlauchy and Kurtzman were included based on multi-locus DNA sequences (Péter et al. 2011; Péter et al. 2012). This was followed by Kurtzman and Robnett (2014) in which eight genera were accepted into Trichomonascaceae while the other anamorphic species such as *Candidaglaebosa* clade of the family are currently members of the polyphyletic genus *Candida* (Lachance et al. 2011; Daniel et al. 2014). The majority of taxa included in the family Trichomonascaceae form septate hyphae, but members of the genus *Wickerhamiella* do not (Kurtzman and Robnett 2007; Lachance and Kurtzman 2011) and instead the genus *Spencermartinsiella* with the type species *Spencermartinsiellaeuropaea* form blastoconidia on small denticles (Péter et al. 2011). With the exception of *Trichomonascusfarinosus* (de Hoog, Rantio-Lehtimäki & M.Th. Smith) Kurtzman & Robnett, all teleomorphic species that form septate hyphae are also heterothallic (Kurtzman and Robnett 2007; Smith et al. 2011a; Péter et al. 2012).

Members of Trichomonascaceae occur on a wide range of substrates in terrestrial and marine environments worldwide (Sakpuntoon et al. 2020), and some have ecological distribution patterns that may imply close relationships with insects. Species have been isolated either directly from insects or insect related substrates. Furthermore, the species of Trichomonascaceae are of economic importance to fields of food production, cosmetics, environment, medicine, and agriculture. For instance, several species of *Blastobotrys* von Klopotek play vital roles in production of lipids (Smith et al. 2011b; Thomas et al. 2019), while some species of *Wickerhamiella* are pathogens of humans (Lachance and Kurtzman 2011; Avchar et al. 2019; Belloch et al. 2020). Additionally, some members of *Sugiyamaella*, including *Su.bahiana* L.M. Sena et al., *Su.bonitensis* L.M. Sena et al., *Su.boreocaroliniensis* (Kurtzman) H. Urbina & M. Blackw, *Su.lignohabitans* (Kurtzman) H. Urbina & M. Blackw, *Su.valenteae* L.M. Sena et al., *Su.xylanicola* Morais, Lachance & Rosa and *Su.xylolytica* L.M. Sena et al., possess the ability to ferment D-xylose into ethanol, and three species: *Su.xylanicola*, *Su.lignohabitans*, and *Su.valenteae* are capable of producing highly active xylanases. (Kurtzman 2011b; Morais et al. 2013a, b; Sena et al. 2017). Therefore, the discovery of novel yeasts in Trichomonascaceae is of both fundamental and applied importance. Moreover, increasing our knowledge and understanding of this group of yeast may provide useful information for their sustainable utilization and conservation of natural resources.

Rotting wood, which contains diverse and abundant assimilable carbon compounds, is known to be a rich habitat for yeast species. In the past few years, thirteen species of Trichomonascaceae, including *Blastobotrys*, *Spencermartinsiella*, and *Sugiyamaella*, were obtained from rotting wood in China, which includes six new species and seven known species (Wang et al. 2010; Guo et al. 2012; Huang et al. 2018; Chai et al. 2020; Shi et al. 2021). Although the samples of rotting wood were collected in a relatively small geographical area in China, the Trichomonascaceae species are diverse in this rich ecological environment.

During extensive investigations on the diversity of yeast inhabiting rotting wood from China, several unknown yeast strains were collected from Henan Province, and their morphology suggested species of *Diddensiella*, *Sugiyamaella*, and *Zygoascus*. To investigate their taxonomy further, phylogenetic analyses, based on combined ITS and nrLSU sequences, were carried out. Both morphological characteristics and molecular evidence demonstrate that these yeasts represent four new species of Trichomonascaceae, which are described here.

## ﻿Materials and methods

### ﻿Sample collection and yeast isolation

Samples of rotting wood were collected in the Tianchi Mountain National Forest Park (34°33'N, 112°28'E) located near Luoyang City, Henan Province, China. The national forest park is at 850 m above sea level (MASL) and has a continental monsoon climate. The average annual temperature is between 14 °C and 16 °C, and the average annual rainfall is greater than 800 mm. Forty samples of decaying wood were collected between September and October in 2020. Samples were stored in sterile plastic bags and transported under refrigeration to the laboratory within 24 hours. Yeast strains were isolated from rotting wood samples according to previously described methods (Huang et al. (2018) and Shi et al. (2021). One gram of each sample was added to 20 mL sterile yeast extract-malt extract (YM) broth (0.3% yeast extract, 0.3% malt extract, 0.5% peptone, 1% glucose, pH 5.0 ± 0.2), supplemented with 0.025% sodium propionate and 200 mg/L chloramphenicol in a 150 mL Erlenmeyer flask, and then cultured for 3–10 days at 180 rpm on a rotary shaker. Subsequently, 0.1 mL aliquots of the enrichment culture and appropriate decimal serial dilutions were plated on YM agar plates and incubated at 25 °C for 3–4 days. Different yeast colony morphotypes were then isolated via repeated plating on YM agar. Isolates were stored on YM agar slants at 4 °C or in 15% glycerol at −80 °C. All isolates were stored in Microbiology Lab of Nanyang Normal University (NYNU; Nanyang, China), and ex-type cultures of novel yeast were deposited in the fungal collection at Westerdijk Fungal Biodiversity Institute (CBS; Utrecht, The Netherlands). Species nomenclature and descriptions were registered in MycoBank (www.mycobank.org, accessed on February 9, 2022).

### ﻿Morphological and physiological investigation

Morphological and physiological properties were determined according to methods previously described in Kurtzman et al. (2011). Carbon and nitrogen assimilation tests were performed using liquid media and growth was observed for up to 4 weeks. Carbon fermentation was tested in yeast extract peptone (YP) base media (1% yeast extract and 2% peptone, pH 5.0 ± 0.2), and Durham tubes were used to visualise carbon dioxide production. Growth rates at a range of temperatures (30 °C, 35 °C, 37 °C, and 40 °C) were assessed by streaking cells on to yeast extract peptone glucose (YPD) agar (1% yeast extract, 2% peptone, 2% glucose, 2% agar, pH 5.0 ± 0.2) plates and incubating them for~2 weeks. Formation of true hyphae and pseudohyphae were investigated using the Dalmau plate method on both cornmeal (CM) and 5% malt extract (ME) agar plates. Induction of the sexual stage was tested by incubating single or mixed cultures of the each of the two strains on PDA agar, cornmeal (CM) agar, 5% malt extract (ME) agar, V8 (1:9) agar, Gorodkowa agar, or yeast carbon base plus 0.01% ammonium sulfate (YCBAS) agar at 25 °C for 2 months (Kurtzman 2011b; Péter et al. 2012; Nagatsuka et al. 2016).

### ﻿DNA amplification and sequencing

Genomic DNA was extracted from each of the yeasts using the Ezup Column Yeast Genomic DNA Purification Kit according to the manufacturer’s protocol (Sangon Biotech, China). The rDNA ITS1-5.8S-ITS2 (ITS) region was amplified using the primer pair ITS1/ITS4 (White et al. 1990). The D1/D2 domain of nrLSU rDNA (nrLSU) was amplified using the primer pair NL1/NL4 (Kurtzman and Robnett 1998). The following parameters were used to amplify the ITS and nrLSU regions: an initial denaturation step of 2 min at 95 °C, followed by 35 cycles of 30 s at 95 °C, 30 s at 51 °C, and 40 s at 72 °C, and a final extension of 10 min at 72 °C (Shi et al. 2021). PCR products were directly purified and sequenced by Sangon Biotech Inc. (Shanghai, China). The identity and quality of the newly-obtained sequences were assessed by comparing them to sequences in GenBank and assembling them with BioEdit (Hall 1999). Sequences were then submitted to GenBank (https://www. ncbi.nlm.nih.gov/genbank/; Table 1).

**Table 1.** DNA sequences used in the molecular phylogenetic analysis.

Species	Strain	Locality	Sample	ITS	D1/D2
*Blastobotrysindianensis*	CBS 9600^T^	USA	White fungus	NR_153638	NG_055333
*Diddensiellacaesifluorescens*	CBS 12613^T^	Hungary	Rotten wood	JF895509	GU195654
*D.santjacobensis*	CBS 8183^T^	USA	Fallen trunk	NR_151808	NG_058985
*D.transvaalensis*	CBS 6663^T^	South Africa	Forest litter	N/A	DQ442702
***D.luoyangensis***	**NYNU 201062^T^**	**China**	**Rotten wood**	**MW374289**	**MW362346**
***D.luoyangensis***	**NYNU 201074**	**China**	**Rotten wood**	**MW374461**	**MW374460**
*Middelhovenomycespetrohuensis*	CBS 8173^T^	Chile	Rotten trunk	NR_156314	NG_055211
*Middelhovenomycestepae*	CBS 5115^T^	Chile	Decaying tepa tree	NR_154200	NG_055181
*Spencermartinsiellacellulosicola*	CBS 11952^T^	China	Rotten wood	NR_151783	NG_055207
*Sp.europaea*	CBS 11730^T^	Hungary	Rotten wood	NR_111481	NG_042528
*Sp.ligniputridi*	CBS 12585^T^	Hungary	Rotten wood	NR_155842	NG_055382
*Sp.silvicola*	CBS 11952^T^	Brazil	Rotting wood	KT222943	KC906243
*Sugiyamaellaamericana*	CBS 10352^T^	USA	Frass	NR_137759	DQ438193
*Su.Ayubii*	CBS 14108^T^	Brazil	Rotting wood	NR_155796	KR184132
*Su.Bahiana*	CBS 13474^T^	Brazil	Rotting wood	NR_155810	KC959941
*Su.Bonitensis*	CBS 14270^T^	Brazil	Rotting wood	NR_155798	KT006004
*Su.Boreocaroliniensis*	NRRL YB-1835^T^	USA	Frass	NR_165963	DQ438221
*Su.Bullrunensis*	CBS 11840^T^	USA	Insect	NR_111543	HM208601
*Su. Castrensis*	NRRL Y-17329^T^	Chile	Rotting wood	NR_111229	DQ438195
*Su.Carassensis*	CBS 14107^T^	Brazil	Rotting wood	NR_155808	KX550111
*Su. Chiloensis*	CBS 8168^T^	Chile	Rotted wood	DQ911454	DQ438217
*Su.Chuxiongensis*	NYNU 181038^T^	China	Rotting wood	MK682800	MK682795
***Su.cylindrica***	**NYNU 201067** ^T^	**China**	**Rotting wood**	**MW368732**	**MW368731**
***Su.Cylindrica***	**NYNU 201034**	**China**	**Rotting wood**	**OM501585**	**OM501589**
*Su. Floridensis*	NRRL YB-3827^T^	USA	Frass	NR_111230	DQ438222
*Su.grinbergsii*	NRRL Y-27117^T^	Chile	Insect	KY102116	DQ438199
*Su. Japonica*	CBS 10354^T^	Japan	Frass	NR_111239	DQ438202
*Su.Ligni*	CBS 13482^T^	Brazil	Rotting wood	KX550112	KX550112
*Su.lignohabitans*	NRRL YB-1473^T^	USA	Decayed log	NR_119622	DQ438198
*Su.marionensis*	NRRL YB-1336^T^	USA	Decayed log	NR_111237	DQ438197
*Su.marilandica*	NRRL YB-1847^T^	USA	Frass	NR_165965	DQ438219
*Su.mastotermitis*	CBS 14182^T^	Berlin	Termite	NR_156606	KU883286
*Su.neomexicana*	CBS 10349^T^	USA	Frass	NR_165966	DQ438201
*Su.novakii*	NRRL Y-27346^T^	Hungary	Rotting wood	NR_111235	DQ438196
*Su.paludigena*	NRRL Y-12697^T^	Russia	Peat	NR_111236	DQ438194
*Su.pinicola*	CBS 10348^T^	USA	Frass	NR_165967	DQ438200
*Su.qingdaonensis*	CBS 11390^T^	China	Rotting wood	NR_151806	FJ613527
***Su.robnettiae***	**NYNU 201066 ^T^**	**China**	**Rotting wood**	**MW368730**	**MW368701**
***Su.robnettiae***	**NYNU 201005**	**China**	**Rotting wood**	**OM501584**	**OM501586**
*Su.smithiae*	CBS 7522.2^T^	Brazil	Soil	DQ911455	DQ438218
*Su.trypani*	CBS 15876^T^	Poland	Soil	MK388412	MK387312
*Su.valdiviana*	NRRL Y-7791^T^	Chile	Rotting wood	NR_111544	DQ438220
*Su.valenteae*	CBS 14109^T^	Brazil	Rotting wood	NR_155797	KT005999
*Su.xiaguanensis*	NYNU 161041^T^	China	Rotting wood	KY213802	KY213817
*Su.xylanicola*	CBS 12683^T^	Brazil	Rotting wood	KC493642	KC493642
*Su.xylolytica*	CBS 13493^T^	Brazil	Rotting wood	KU214874	KF889433
*Su.yunnanensis*	NYNU 161059^T^	China	Rotting wood	MT257259	MT257257
*Tortisporaganteri*	CBS 12581^T^	Mexico	Necrotic plant tissue	NR_154483	KC681893
*Tortisporacaseinolytica*	CBS 7781^T^	USA	Necrotic plant tissue	NR_154482	NG_055343
*Trichomonascuspetasosporus*	CBS 9602^T^	USA	Frass	NR_155940	NG_055332
*Zygoascusbiomembranicola*	CBS 14157^T^	Japan	Viscous gel	NR_156007	LC060997
*Z.bituminiphila*	CBS 8813^T^	Canada	Tar	NR_137545	NG_055308
*Z.hellenicus*	CBS 5839^T^	Germany	Mastitic bovine udder	NR_111258	NG_055323
*Z.meyerae*	CBS 4099^T^	Greece	Fermenting grape must	AY447022	DQ438189
*Z.ofunaensis*	CBS 8129^T^	Japan	Soil	N/A	NG_066348
*Z.polysorbophila*	CBS 7317^T^	Japan	Viscous gel	NR_160311	NG_064312
*Z.tannicola*	CBS 6065^T^	France	Vegetable tanning fluid	KY106018	NG_058446
***Z.detingensis***	**NYNU 201087^T^**	**China**	**Rotting wood**	**MW374088**	**MW368733**
***Z.detingensis***	**NYNU 201011**	**China**	**Rotting wood**	**OM501590**	**OM501591**

Notes: Metabolically inactive ex-type strains are indicated by “T” after the species name; “N/A” means that sequences were not available; Bold indicates strains that were isolated in this study.

### ﻿Phylogenetic analyses

Species in the family Trichomonascaceae with high similarity to the new species described here were selected as references in the phylogenetic analyses. *Tortisporacaseinolytica*CBS 7781^T^ and *Tor.ganteri*CBS 12581^T^ were used as outgroup. NCBI accession numbers of sequences used in the phylogenetic tree are listed in Table 1. Initial alignment of the combined ITS + nrLSU dataset was performed using the online version MAFFT 6.0 (Katoh and Toh 2010) followed by manual evaluations and adjustments in BioEdit as needed to obtain reliable and high quality results (Hall 1999). The best-fit nucleotide substitution models for separate and combined nucleotide sequences were selected using jModelTest v2.1.7 (Darriba et al. 2012) according to the Akaike Information Criterion (AIC). The final concatenated sequence alignment was deposited in TreeBase (http://www.treebase.org; submission ID S29358).

Maximum likelihood (ML) and Bayesian inference (BI) analyses were used for the phylogenetic analyses. The ML analysis was carried out using RAxmL v.7.2.8 with a GTR + G + I, model of site substitution including estimation of Gamma-distributed rate heterogeneity and a proportion of invariant sites (Stamatakis 2006). Branch support was evaluated using bootstrapping with 1000 replicates (Hillis and Bull 1993). The BI analysis was performed using MrBayes v3.2 (Ronquist et al. 2012), for two independent runs, each with four Markov chains Monte Carlo (MCMC) independent runs for 5 ×10^6^ generations (split frequencies = 0.011). The first 25% of trees were discarded as “burn-in” of each analysis and the remaining 75% were then used to calculate Bayesian posterior probabilities of the majority rule consensus tree.

Phylogenetic trees from the ML and BI analyses were visualised with FigTree v1.4.3 (Rambaut 2016) and edited in Adobe Illustrator CS6. Branches that received bootstrap support for maximum likelihood (BS) and Bayesian posterior probabilities (BPP) greater than or equal to 50% (BS) and 0.95 (BPP) were considered to be significantly supported.

## ﻿Results

### ﻿Molecular phylogenetic analysis

**Figure 1.**
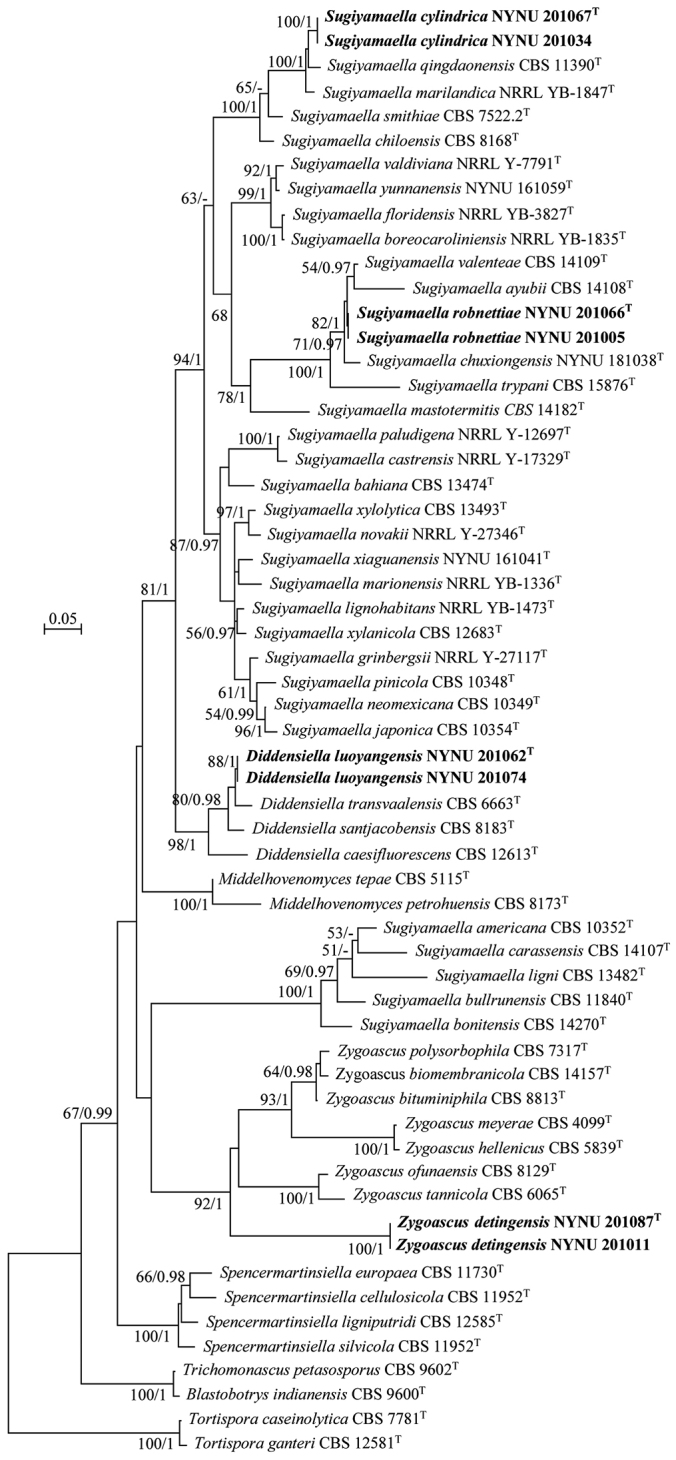
Maximum-likelihood phylogenetic tree based on ITS and nrLSU nucleotide sequences. Bootstrap values (BP) ≥ 50% from ML analysis and Bayesian posterior probabilities (BPP) ≥ 0.95 are shown on the branches. Newly described species are indicated in bold and their metabolically inactive ex-type strains are indicated by “T” after the species name.

The combined ITS and nrLSU dataset was analysed to infer the phylogenetic relationships of the family Trichomonascaceae and the new Chinese isolates. The dataset consisted of 59 sequences including the outgroup, *Tortisporacaseinolytica*CBS 7781^T^ and *Tor.ganteri*CBS 12581^T^. A total of 943 characters including gaps (376 for ITS and 567 for nrLSU) were included in the phylogenetic analysis. GTR + I + G was inferred as the best-fit model for the combined nrLSU and ITS nucleotide sequences according to the AIC in jModelTest v2.1.7 (Darriba et al. 2012). The topologies of the phylogenetic tree of ML and BI analyses are identical, and only the ML tree with a final optimisation likelihood value of –12097.50 is shown in Fig. 1. RAxML bootstrap support values (BS) ≥ 50% and Bayesian posterior probability values (BPP) ≥ 0.95 are shown above the branches and indicated with bolded lines.

In the phylogeny (Fig. 1), newly generated strains in this study nested in the genera *Diddensiella*, *Sugiyamaella*, and *Zygoascus* within the Trichomonascaceae. *D.luoyangensis* clustered in the *Diddensiella* clade with an affinity to *D.santjacobensis* (C. RamÍrez & A. González) Péter, Dlauchy & Kurtzman and *D.transvaalensis* (Kurtzman) Péter, Dlauchy & Kurtzman. *Su.cylindrica* and *Su.robnettiae* clustered in the *Sugiyamaella* clade with close similarity to the type species *Su.smithiae* (Giménez-Jurado) Kurtzman and Robnett (2007), and to other related species with high bootstrap support (BS = 94%; BPP = 1.0). Additionally, *Su.cylindrica* clustered together with *Su.marilandica* (Kurtzman) H. Urbina & M. Blackw and *Su.qingdaonensis* (F.L. Li & S.A. Wang) Handel, Wang, Yurkov & König with strong bootstrap support (BS 100%, BPP 1.0), while *Su.robnettiae* formed a separate lineage within *Sugiyamaella* that included *Su.ayubii* L.M. Sena et al., *Su.chuxiongensis* C.Y. Chai & F.L. Hui, and *Su.valenteae* L.M. Sena et al. *Z.detingensis* formed a unique branch of the tree which was clearly distinct and diverged from other species of *Zygoascus*.

### ﻿Taxonomy

#### ﻿ Diddensiella luoyangensis

Taxon classificationFungiSaccharomycetalesTrichomonascaceae

C.Y. Chai & F.L. Hui sp. nov.

227D4E90-E7B9-59A7-AD87-310FD1CA4E4A

 842899

Fig. 2

##### Etymology.

The specific epithet *luoyangensis* refers to the geographic origin of the type strain: Luoyang City, Henan.

**Figure 2.**
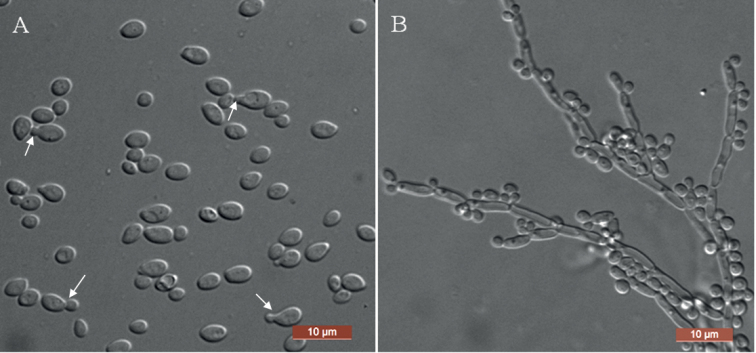
Morphology of *D.luoyangensis* (NYNU 201062, holotype) **A** budding cells were indicated by arrows in YM broth after 3 d **B** pseudohyphae and true hyphae on CM agar after 14 d. Scale bars: 10 μm.

##### Type.

China, Henan Province, Luoyang City, Song County, the Tianchi Mountain National Forest Park, in rotting wood, October 2020, J.Z. Li & Z.T. Zhang (holotype NYNU 201062^T^, ex-type CBS 16659 = CICC 33512, holotype and ex-type are preserved in a metabolically inactive state).

##### Description.

In YM broth after 3 days at 25 °C, cells are ovoid (2–3 × 3–5 μm) and occur singly or in pairs. Budding is multilateral. Sediment is formed after a month, but a pellicle is not observed. On YM agar after 3 days at 25 °C, colonies are white to cream- coloured, convex, butyrous, and smooth with entire margins. In Dalmau plate culture on corn meal agar, pseudohyphae and true hyphae are formed. Asci or signs of conjugation are not observed on sporulation media. Fermentation of sugars is absent. Glucose, galactose, l-sorbose, glucosamine, d-ribose, d-xylose, l-arabinose, d-arabinose, l-rhamnose, sucrose, maltose, trehalose, methyl α- d-glucoside, cellobiose, salicin, melibiose, lactose, raffinose, melezitose, inulin, glycerol, erythritol, ribitol, d-glucitol, d-mannitol, galactitol, *myo*-inositol, d-glucono-1, 5-lactone, 2-keto-d-gluconate, 5-keto-d-gluconate, d-gluconate, d-glucuronate, dl-lactate succinate, citrate, and ethanol are assimilated as sole carbon sources. Methanol is not assimilated. l-lysine, creatine, glucosamine, and d-tryptophan are assimilated as sole nitrogen sources, while nitrate, nitrite, ethylamine, cadaverine, creatinine, and imidazole are not assimilated. Minimum growth temperature is 15 °C, and maximum growth temperature is 37 °C. Growth in the presence of 0.1% cycloheximide is present, but growth in the presence of 10% NaCl plus 5% glucose and 1% acetic acid is absent. Starch-like compounds are not produced. Urease activity and diazonium blue B reactions are negative.

##### Additional isolate examined.

China, Henan Province, Luoyang City, Song County, the Tianchi Mountain National Forest Park, in rotting wood, October 2020, J.Z. Li & Z.T. Zhang (NYNU 201074).

##### Notes.

Two strains were collected from two different substrates, representing *D.luoyangensis*, clustered in the *Diddensiella* clade which is sister to species *D.transvaalensis*. *D.luoyangensis* differed from *D.transvaalensis* by 1.6% substitutions in the D1/D2 domain. Furthermore, we were unable to align the ITS sequence of *D.luoyangensis* with the *D.transvaalensis* type strain, because the ITS sequence of *D.transvaalensis* is not currently available from either the NCBI GenBank or CBS databases. Physiologically, *D.luoyangensis* differs from its closely related species, *D.transvaalensis* (Lachance et al. 2011), based on growth in l-rhamnose, lactose, inulin, d-gluconate and growth at 37 °C, which are present for *D.luoyangensis* and absent for the latter species. Moreover, *D.transvaalensis* ferments glucose and galactose, while this new species does not.

#### ﻿ Sugiyamaella cylindrica

Taxon classificationFungiSaccharomycetalesTrichomonascaceae

C.Y. Chai & F.L. Hui sp. nov.

57BF658E-C9D5-58BC-82D8-7077A00BF9EE

 842900

Fig. 3

##### Etymology.

The specific epithet *cylindrica* refers to the cylindrical vegetative cells of the type strain.

**Figure 3.**
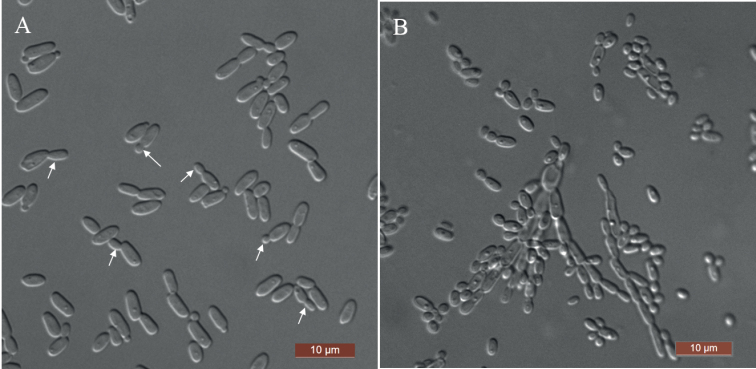
Morphology of *Su.cylindrica* (NYNU 201067, holotype) **A** budding cells were indicated by arrows in YM broth after 3 d **B** rudimentary pseudohyphae on CM agar after 14 d. Scale bars: 10 μm.

##### Type.

China, Henan Province, Luoyang City, Song County, the Tianchi Mountain National Forest Park, in rotting wood, October 2020, J.Z. Li & Z.T. Zhang (holotype NYNU 201067^T^, ex-type CBS 16662 = CICC 33514, holotype and ex-type are preserved in a metabolically inactive state).

##### Description.

In YM broth after 3 days at 25 °C, cells are cylindrical (2–3 × 5–7 μm) and occur singly or in pairs. Budding is multilateral. Sediment is formed after a month, but a pellicle is not observed. On YM agar after 3 days at 25 °C, colonies are white to cream-coloured, butyrous, convex and smooth with entire margins. In Dalmau plate culture on corn meal agar, rudimentary pseudohyphae are formed. Asci or signs of conjugation are not observed on sporulation media. Glucose and trehalose are weakly fermented, but, galactose, maltose sucrose, melibiose, lactose, cellobiose, melezitose, raffinose, inulin and xylose are not fermented. Glucose, galactose, l-sorbose, glucosamine, d-ribose, d-xylose, l-arabinose, d-arabinose, l-rhamnose, sucrose, maltose, trehalose, methyl α-d-glucoside, cellobiose, salicin, melibiose, raffinose, melezitose, inulin, glycerol, erythritol, ribitol, d-glucitol, d-mannitol, galactitol, *myo*-inositol, d-glucono-1, 5-lactone, 2-keto-d-gluconate, 5-keto-d-gluconate, d-glucuronate, dl-lactate succinate, and ethanol are assimilated as sole carbon sources. Lactose, d-gluconate, citrate and methanol are not assimilated. Nitrate, nitrite, l-lysine, creatine, glucosamine, and d-tryptophan are assimilated as sole nitrogen sources. Ethylamine, cadaverine, creatinine, and imidazole are not assimilated. Minimum growth temperature is 15 °C, and maximum growth temperature is 35 °C. Growth in the presence of 0.1% cycloheximide is present, but growth in the presence of 1% acetic acid and 10% NaCl plus 5% glucose is absent. Starch-like compounds are not produced. Urease activity and diazonium blue B reactions are negative.

##### Additional isolate examined.

China, Henan Province, Luoyang City, Song County, the Tianchi Mountain National Forest Park, in rotting wood, October 2020, J.Z. Li & Z.T. Zhang (NYNU 201034).

##### Notes.

Two strains were collected from two different substrates, representing *Su.cylindrica*, clustered in the *Sugiyamaella* clade and are closely related to *Su.marilandica* and *Su.qingdaonensis*. The nucleotide differences between the new species and the close relatives *Su.marilandica* and *Su.qingdaonensis* are 1.1–1.4% substitutions in the D1/D2 domain and 5.0–5.9% substitutions in the ITS region, respectively. Physiologically, *Su.cylindrica* differs from the closely related species *Su.marilandica* and *Su.qingdaonensis* (Wang et al. 2010; Kurtzman 2011b) in its ability to assimilate glycerol and dl-lactate and to grow at 35 °C. Additionally, the new species ferments trehalose, while *Su.marilandica and Su.qingdaonensis* do not.

#### ﻿ Sugiyamaella robnettiae

Taxon classificationFungiSaccharomycetalesTrichomonascaceae

C.Y. Chai & F.L. Hui sp. nov.

89B11835-90B0-5C10-9A56-7264B5C9DB71

 842901

Fig. 4

##### Etymology.

The specific epithet *robnettiae* named in honour of Christie J. Robnett for her proposal of the genus *Sugiyamaella*.

**Figure 4.**
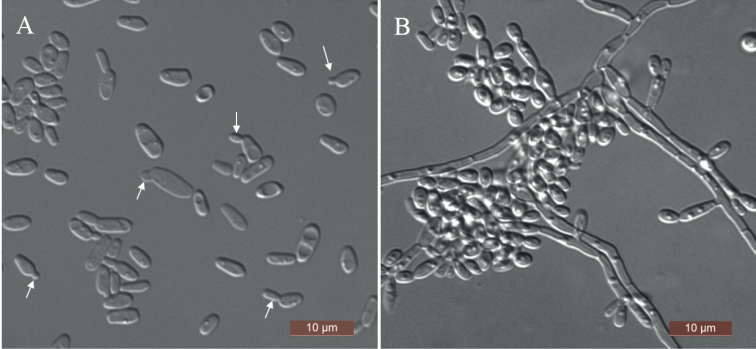
Morphology of *Su.robnettiae* (NYNU 201066, holotype) **A** budding cells were indicated by arrows in YM broth after 3 d **B** pseudohyphae and true hyphae on CM agar after 14 d. Scale bars: 10 μm.

##### Type.

China, Henan Province, Luoyang City, Song County, the Tianchi Mountain National Forest Park, in rotting wood, October 2020, J.Z. Li & Z.T. Zhang (holotype NYNU 201066^T^, ex-type CBS 16663 = CICC 33513, holotype and ex-type are preserved in a metabolically inactive state).

##### Description.

In YM broths after 3 days at 25 °C, the cells are ellipsoidal to elongate (2–4 × 2–8 μm) and occur singly or in pairs. Budding is multilateral. Sediment is formed after a month, but a pellicle is not observed. On YM agar after 3 days at 25 °C, colonies are white to cream-coloured, convex, buttery and smooth with entire margins. In Dalmau plate culture on corn meal agar, pseudohyphae and true hyphae are formed. Asci or signs of conjugation are not observed on sporulation media. Fermentation of sugars is absent. Glucose, galactose, l-sorbose, glucosamine, d-xylose, l-arabinose, d-arabinose, l-rhamnose, sucrose, maltose, trehalose, methyl α-d-glucoside, cellobiose, salicin, arbutin, lactose, inulin, glycerol, erythritol, ribitol, xylitol, d-glucitol, d-mannitol, galactitol, d-glucono-1, 5-lactone, 2-keto-d-gluconate, 5-keto-d-gluconate, succinate, citrate, and ethanol are assimilated as sole carbon sources. d-ribose, melibiose, raffinose, melezitose, *myo*-inositol, d-gluconate, dl-lactate, and methanol are not assimilated. Nitrate, nitrite, creatine, glucosamine, and d-tryptophan are assimilated as sole nitrogen sources. Ethylamine, l-lysine, creatinine, and imidazole are not assimilated. Minimum growth temperature is 15 °C, and maximum growth temperature is 35 °C. Growth in the presence of 0.1% cycloheximide is present, but growth in the presence of 10% NaCl plus 5% glucose and 1% acetic acid is absent. Starch-like compounds are not produced. Urease activity and diazonium blue B reactions are negative.

##### Additional isolates examined.

China, Henan Province, Luoyang City, Song County, the Tianchi Mountain National Forest Park, in rotting wood, October 2020, J.Z. Li & Z.T. Zhang (NYNU 201005).

##### Notes.

Two strains were collected from two different substrates, formed a well-supported group related to *Su.chuxiongensis*, representing a new species, *Su.robnettiae. Su.robnettiae* differs from *Su.chuxiongensis* by 1.9% substitutions in the D1/D2 domain and 6.4% substitutions in the ITS region. Physiologically, unlike *Su.chuxiongensis* (Shi et al., 2021), *Su.robnettiae* is unable to assimilate d-ribose, melibiose, raffinose, or melezitose but is able to assimilate glycerol and lactose.

#### ﻿ Zygoascus detingensis

Taxon classificationFungiSaccharomycetalesTrichomonascaceae

C.Y. Chai & F.L. Hui sp. nov.

AE962363-835B-5555-9B02-21A34DC73E84

 842902

Fig. 5

##### Etymology.

The specific epithet *detingensis* refers to the geographic origin of the type strain, Deting Town, Henan.

**Figure 5.**
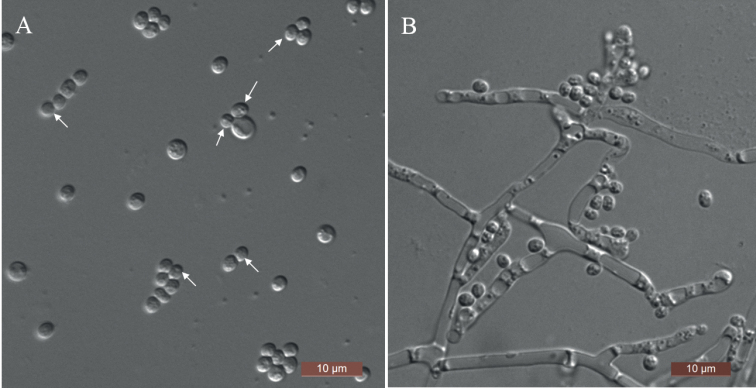
Morphology of *Z.detingensis* (NYNU 201087, holotype) **A** budding cells were indicated by arrows in YM broth after 3 d **B** pseudohyphae and true hyphae on CM agar after 14 d. Scale bars: 10 μm.

##### Type.

China, Henan Province, Luoyang City, Song County, the Tianchi Mountain National Forest Park, in rotting wood, October 2020, J.Z. Li & Z.T. Zhang (holotype NYNU 201087 ^T^, ex-type CBS 16667 = CICC 33516, holotype and ex-type preserved in a metabolically inactive state).

##### Description.

In YM broth after 3 days at 25 °C, cells are subglobosal to globosal (2–3 × 2–4 μm) and occur singly or in pairs. Budding is multilateral. Sediment is formed after a month, but a pellicle is not observed. On YM agar after 3 days at 25 °C, colonies are cream, smooth, opalescent, convex and glistening. In Dalmau plate culture on corn meal agar, pseudohyphae and true hyphae are formed. Asci or signs of conjugation are not observed on sporulation media. Fermentation of sugars is absent. Glucose, galactose (weak), glucosamine, d-ribose (weak), d-xylose, d-arabinose (weak), l-arabinose (weak), l-rhamnose (weak), sucrose (weak), maltose (weak), trehalose, methyl α-d-glucoside (weak), cellobiose (weak), salicin, melibiose, lactose (weak), raffinose, melezitose (weak), inulin (weak), glycerol (weak), erythritol, ribitol (weak), xylitol (weak), d-glucitol (weak), d-mannitol (weak), galactitol (weak), *myo*-inositol (weak), d-glucono-1, 5-lactone, 2-keto-d-gluconate, d-gluconate (weak), d-glucuronate (weak), dl-lactate (weak), succinate (weak), and ethanol are assimilated as sole carbon sources. l-sorbose, citrate, and methanol are not assimilated. Ethylamine, glucosamine, and l-lysine are assimilated as sole nitrogen sources. Nitrate, nitrite, cadaverine, creatine, creatinine, imidazole, and d-tryptophan are not assimilated. Minimum growth temperature is 15 °C, and maximum growth temperature is 37 °C. Growth in the presence of 0.1% cycloheximide is present, but growth in the presence of 10% NaCl plus 5% glucose and 1% acetic acid is absent. Starch-like compounds are not produced. Urease activity and diazonium blue B reactions are negative.

##### Additional isolate examined.

China, Henan Province, Luoyang City, Song County, the Tianchi Mountain National Forest Park, in rotting wood, October 2020, J.Z. Li & Z.T. Zhang (NYNU 201011).

##### Notes.

Two strains were collected from two different substrates, both representing *Z.detingensis*, branched separately from the *Zygoascus* clade. *Z.detingensis* differed from the other *Zygoascus* species by more than 9.7% substitutions in the D1/D2 domain and 11.5% substitutions in the ITS region, respectively. Physiologically, *Z.detingensis* differs from its closely related species, *Z.bituminiphila* (V. Robert, B. Bonjean, Karutz, Paschold, W. Peeters & Wubbolts) Nagatsuka, Kiyuna & Sugiyama (Nagatsuka et al. 2016), in its inability to assimilate l-sorbose and its ability to assimilate l-rhamnose, methyl α-d-glucoside, melibiose, lactose, inulin melezitose, erythritol, and 2-keto-d-gluconate. Moreover, *Z.bituminiphila* ferments glucose, galactose, trehalose, and cellobiose, while *Z.detingensis* does not.

## ﻿Discussion

In the present study, we collected rotting wood from the Tianchi Mountain National Forest Park located near Luoyang City in Henan Province of China. From these samples, we isolated several yeast strains. Some of these yeasts are known species, such as *Metschnikowiahenanensis*, *Saturnisporagalanensis*, *Wickerhamomycesmenglaensis* and *Deakozymayunnanensis*. Here, we recovered eight isolates from eight rotting woods of *Trichomonascaceae* yeast representing four new species belonging to the genera *Diddensiella*, *Sugiyamaella*, and *Zygoascus*. We described these new species as *D.luoyangensis*, *Su.cylindrica*, *Su.Robnettiae*, and *Z.detingensis* based on molecular phylogenetic and morphological evidence. A thorough and comprehensive phylogenetic analysis of the family *Trichomonascaceae* based on the combined ITS and the D1/D2 domains of the LSU rRNA gene sequences is provided, including almost all GenBank representatives and newly generated sequences, which may serve as a reference for the field. This study provides information on the species delimitation of the family *Trichomonascaceae* based on morphological and phylogenetic evidence.

Our phylogenetic analyses, based on ITS and the D1/D2 domains of the LSU rRNA gene sequences, are in concordance with previous studies (Morais et al. 2013b; Sena et al. 2017; Shi et al. 2021). However, the genus *Sugiyamaella* of *Trichomonascaceae* is not a monophyletic group. Morais et al. (2013b) indicated that *Sugiyamaella* is polyphyletic, where the species are intertwined with representatives of the genera *Diddensiella* and *Spencermartinsiella*. From the latter study, the genus could be divided into two main clades, which were later supported by Sena et al. (2017) and Shi et al. (2021). In this study, all species of *Sugiyamaella* and related genera were used to refine our understanding of the evolutionary relationships of this family, based on the ITS and nrLSU dataset. As shown in Fig. 1, all genera of *Trichomonascaceae* formed monophyletic groups with the exception of *Sugiyamaella* in which two main clades were reconstructed: (i) *Su.smithiae* (the type species), *Su.lignohabitans*, and *Su.valdiviana* and their related species and (ii) *Su.americana*, *Su.bullrunensis*, (S.O. Suh, Houseknecht & J.J. Zhou) H. Urbina & M. Blackw, *Su.carassensis* L.M. Sena et al. and *Su.ligni* L.M. Sena et al.

In recent years, more than 40 yeast species have been identified from rotting wood in China (Wang et al. 2010; Guo et al. 2012; Gao et al. 2017; Zheng et al. 2017; Huang et al. 2018; Chai et al. 2020; Lv et al. 2020; Shi et al. 2021). Among them, at least 16 species of *Trichomonascaceae* have been isolated from rotting wood in China, including six new species previously obtained from China (*Bla.xishuangbannaensis*, *Sp.cellulosicola*, *Su.qingdaonensis*, *Su.xiaguanensis*, *Su. Chuxiong*, and *Su.yunanensis*) (Wang et al. 2010; Guo et al. 2012; Huang et al. 2018; Chai et al. 2020; Shi et al. 2021), new records of six species not known to occur in China (*Su.americana*, *Su.ayubii*, *Su.novakii*, *Su.paludigena*, *Su.Valenteae*, and *Su.valdiviana*) (Shi et al. 2021), and four novel species identified in this study (*D.luoyangensis*, *Su.cylindrica*, *Su.robnettiae*, and *Z.detingensis*). In China, there remain species to be discovered, such as those sequences of the D1/D2 domains of the LSU rRNA gene listed under GenBank accessions JN581115 and JN581116. To date, including the four new species described in this study, there are more than 100 species of *Trichomonascaceae* worldwide (www.mycobank.org). Although the taxonomy of *Trichomonascaceae* has been a focus of research in the past, many regions are under-sampled and more novel indigenous *Trichomonascaceae* species will undoubtedly be discovered in the future.

## Supplementary Material

XML Treatment for Diddensiella luoyangensis

XML Treatment for Sugiyamaella cylindrica

XML Treatment for Sugiyamaella robnettiae

XML Treatment for Zygoascus detingensis
